# Effects of prey density and flow speed on plankton feeding by garden eels: a flume study

**DOI:** 10.1242/jeb.243655

**Published:** 2022-04-22

**Authors:** Kota Ishikawa, Heng Wu, Satoshi Mitarai, Amatzia Genin

**Affiliations:** 1 Marine Biophysics Unit, Okinawa Institute of Science and Technology Graduate University, Onna, Okinawa, Japan, 904-0495; 2 The Interuniversity Institute for Marine Sciences in Eilat and Department of Ecology, Evolution and Behavior, The Hebrew University of Jerusalem, Eilat 88103, Israel

**Keywords:** Functional response, Fish, Coral reef, Zooplanktivory, Biomechanics

## Abstract

Feeding by zooplanktivorous fish depends on their foraging movements and the flux of prey to which they are exposed. While prey flux is a linear function of zooplankton density and flow speed, those two factors are expected to contribute differently to fish movements. Our objective was to determine the effects of these factors for garden eels, stationary fish that feed while anchored to the sandy bottom by keeping the posterior parts of their bodies inside a burrow. Using a custom-made flume with a sandy bottom, we quantified the effects of prey density and flow speed on feeding rates by spotted garden eels (*Heteroconger hassi*). Feeding rates increased linearly with prey density. However, feeding rates did not show a linear relationship with flow speed and decreased at 0.25 m s^−1^. Using label-free tracking of body points and 3D movement analysis, we found that the reduction in feeding rates was related to modulation of the eel's movements, whereby the expected increase in energy expenditure was avoided by reducing exposure and drag. No effects of flow speed on strike speed, reactive distance or vectorial dynamic body acceleration (VeDBA) were found. A foraging model based on the body length extended from the burrow showed correspondence with observations. These findings suggest that as a result of their unique foraging mode, garden eels can occupy self-made burrows in exposed shelter-free sandy bottoms where they can effectively feed on drifting zooplankton.

## Abstract (precis)

**Summary:** Analysis of feeding by anchored garden eels in a flume shows their unique strategies, modulating dependence on burrows and foraging movements, to enable effective feeding at a wide range of flow speeds.

## INTRODUCTION

Analysis of behavioral responses of fish to environmental factors is essential to understand their adaptations, habitat use and ecological interactions. Interactions between feeding behavior and environment, in particular, have been studied extensively because feeding is essential to survival (Stoner, 2004).

Zooplankton feeding by fish depends on various biotic and abiotic factors, such as currents (Clarke et al., 2009; Finelli et al., 2009; Fulton et al., 2005; Kiflawi and Genin, 1997), prey density (Kiflawi and Genin, 1997; Noda et al., 1992), prey size (Hill and Grossman, 1993; Manatunge and Asaeda, 1998), light (Howard and Bori, 1972; Manatunge and Asaeda, 1998; Rickel and Genin, 2005), temperature (Nilsson et al., 2010) and risk of predation (Morgan, 1988).

Among those factors, prey density and current speed exhibit relatively high levels of spatiotemporal variability (Folt and Burns, 1999; Fulton and Bellwood, 2005; Johansen, 2014; Reidenbach et al., 2006). Because prey density fluctuates, it is one of the main determinants of foraging strategies. The relationship between prey density and feeding rate is called the functional response, the most typical type of which is described by the disk equation:
(1)

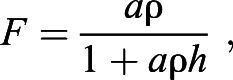
where *F* is the feeding rate, *a* is the search efficiency or attack rate, ρ is prey density and *h* is handling time (Holling, 1959; Solomon, 1949). This is a type II functional response showing that feeding rate increases with prey density at a decelerating rate and plateaus at high prey density, assuming a constant volume is searched per unit time. When handling time is negligible, feeding rate is proportional to prey density. At high prey density, feeding rate plateaus and can be approximated as 1/*h*.

Flow is another parameter that affects feeding among zooplanktivorous fish, as higher prey fluxes occur under stronger flows (Kiflawi and Genin, 1997). Flow can also alter fish behavior and metabolism. Some reef fish optimize their behavior in response to changes in flow conditions by adjusting their strike distance and lateral angle, by changing proportions of fin type usage, or by adopting sheltering behavior, all of which affect search efficiency or handling time (Heatwole and Fulton, 2013; Johansen et al., 2008; Kiflawi and Genin, 1997). In metabolic studies, flow speed influences the energy consumption of fish for locomotion (Fausch, 1984). Although prey density and flow speed contribute linearly to prey flux, flow speed may have a greater impact on fish behavior, via its effect on swimming behavior and strikes (Kiflawi and Genin, 1997; O'Brien et al., 2001; Piccolo et al., 2008). Flumes, in which flow speed and prey density can be precisely controlled, provide the means to differentiate the effects of these two parameters and their causes.

Understanding energy intake and expenditure led to the development of foraging models, which are crucial for predicting adaptation, habitat selection and predator–prey interactions (Kiflawi and Genin, 1997; Piccolo et al., 2014; Rosenfeld and Boss, 2001). Environmental factors therefore alter foraging models as a result of their effects on prey detection and prey capture ability. For drift-feeding river fish and coral reef fish, foraging models are usually based on wedge-shaped reactive volumes calculated from experimentally derived reactive distance and angle (Fausch, 1984; Kiflawi and Genin, 1997). Empirical models and experimental data indicate that in planktivorous reef fish, feeding rate increases and plateaus as prey density increases, while it shows a dome-shaped curve as flow speed increases (Clarke et al., 2009; Kiflawi and Genin, 1997).

Studies on foraging responses to flow speed and prey density have been largely limited to fish that swim freely while foraging for zooplankton in seas, lakes and rivers. Little is known about stationary fish that forage while anchored to the bottom, such as garden eels (Khrizman et al., 2018). Garden eels are zooplanktivorous fish found in tropical sandy habitats, usually near coral reefs. During foraging, the eel's posterior body remains buried in the sand, serving as an anchor. Thirty-six species of garden eel are distributed in the tropics worldwide (Fricke et al., 2021). Because their movements are limited as a consequence of being constrained by their burrows (Smith, 1989), it is useful to understand the relationship between their behavior and the environment. In a field study in the Red Sea, one species of garden eel (*Gorgasia sillneri*) showed a monotonic increase in feeding rate with flow speed, as well as with prey density (Khrizman et al., 2018). Khrizman et al. (2018), suggested that a unique body posture at high flow speed reduces the drag imposed on the eels, avoiding a decrease in feeding rate at high flow speeds. However, *in situ* observations do not allow clear resolution of the separate effects of flow speed and prey density. Precise measurement in a custom-built flume is necessary to resolve the effects of each environmental factor with fine resolution.

The goals of this study were (1) to resolve the effects of prey density and flow speed on garden eel feeding rates in a custom-built flume, (2) to understand the responses of foraging movements and energy consumption using three-dimensional video analysis, and (3) to establish the first foraging model for garden eels. Accomplishing these goals, we compared the results with findings for freely swimming fish and other species of garden eels to find foraging features specific to garden eels.

## MATERIALS AND METHODS

**Fig. 1.**
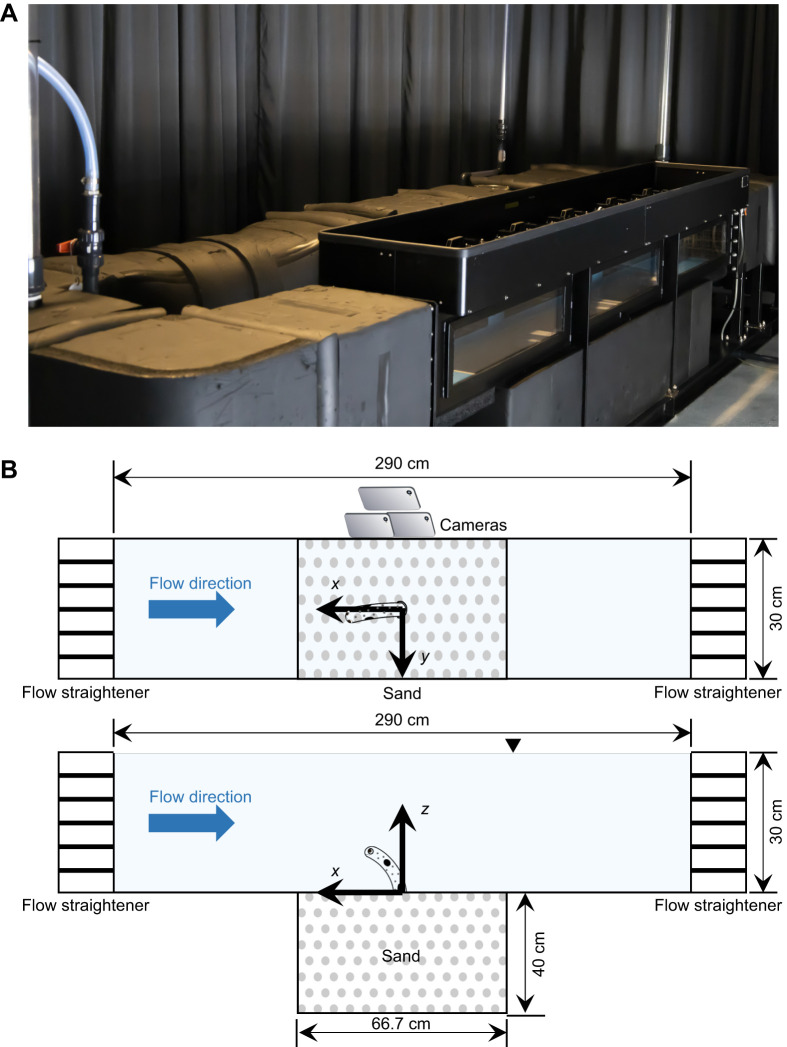
**Flume and test section.** (A) Flume. (B) Schematic diagram of the test section. Upper and lower panels show the top and side views, indicating the location of the garden eel and the three cameras.

Our experiments were conducted in a sandy-bottomed flume at the Marine Science Station of the Okinawa Institute of Science and Technology (Fig. 1). The flume (built by West Japan Fluid Engineering Laboratory Co., Nagasaki, Japan) was a horizontal recirculating open channel with a rectangular cross-section. The test section was 290 cm long, 30 cm wide and 30 cm high. Flow straighteners positioned upstream and downstream of the test section effectively eliminated secondary flows. At the bottom center of the test section, we constructed a pit 66.7 cm long, 30 cm wide and 40 cm deep that was filled with marine sediment in which the eels readily dug their burrows near the center. An impeller drove the water and its frequency controlled the flow speed. Water temperature was maintained at 25±0.5°C by a temperature control unit separated from the flume. Three lights (SPECTRA SP200, Blue Harbor) were placed above the experimental section to create a depth-optimized spectrum adjusted to 20 m between 05:00 h and 19:00 h (14 h:10 h light:dark) with a gradual change of light intensity during the first and last hour.

Garden eels, *Heteroconger hassi* (Klausewitz and Eibl-Eibesfeldt 1959), a species that occurs in the Indo-Pacific Ocean and is common in Okinawa, were purchased from Aqua Planning Co., Ltd. Identity of the species was confirmed by examining the color pattern. Five garden eels, designated A to E, with total lengths of 24.1, 18.0, 30.5, 20.8 and 22.3 cm, respectively, were used in our experiments. Day-old brine shrimp nauplii (*Artemia salina*), 0.59±0.05 mm (mean±s.d., *n*=60) in length were used as the prey. Up to five trials using different prey densities and flow speeds, randomly assigned, were carried out during each working day between 09:00 h and 17:00 h (ZT4–ZT12). During ≥5 days of acclimation prior to the onset of trials, the designated eel was fed *ad libitum* under a flow speed of 0.10 m s^−1^. All garden eel experiments were conducted with approval from the Animal Care and Use Committee at Okinawa Institute of Science and Technology Graduate University.

There were two experimental designs (I and II) to examine the effects of prey density and flow speed. In design I, the effects of prey density were examined for 5 individual eels (A–E) under five levels of prey density (100, 200, 300, 600 and 1000 m^−3^) and two flow speeds (0.10 and 0.20 m s^−1^). In design II, effects of flow speed were examined for the same individuals under four flow speeds (0.10, 0.15, 0.20 and 0.25 m s^−1^) and two levels of prey density (300 and 1000 m^−3^). The range of prey densities was decided based on the range observed in reef environments (Holzman et al., 2005; Khrizman et al., 2018; Kingsford and MacDiarmid, 1988) and that of flow speed was decided based on observations around Okinawan reefs (Rintoul, M. S., Courtney, T. A., Dohner, J. L., Giddings, S. N., Kekuewa, S. A. H., Mitarai, S., Monismith, S. G., Pezner, A. K., and Andersson, A. J., unpublished observation). Two replicates were carried out for each eel for each combination of flow speed and prey density (total *N*=180). Foraging movements of three eels (A, B, C) were analyzed using 3D videography.

### Experimental procedure

We defined the time it took for water to circulate once through the recirculating flume based on the average flow speed as one water cycle in this experiment. The designated flow speed was set for ≥30 min prior to the start of a trial and a custom-made 100 µm plankton net with a square frame, tightly fitting the flume's cross-section, was used to remove particles from the water in the flume during ≥10 cycles. The water temperature control was turned off and valves were closed to stop the inflow and outflow of fresh seawater, so that seawater simply recirculated in the flume. Given the 1.24 m^3^ volume of the flume, individual live prey were manually counted to obtain the target prey density in each trial. Prey were gradually released into the flume during one water cycle followed by ≥5 cycles to thoroughly mix the prey and flume water so as to achieve a nearly homogeneous prey distribution. Garden eels always remained inside the burrow during these preparatory steps, usually emerging from their burrow and starting to feed soon after the end of the final mixing. An eel was allowed to feed for precisely one cycle of the flume to maintain a fixed prey flux despite predation. At the end of the trial, the eel was chased into its burrow by inserting a long stick into the water, causing it to stop feeding immediately. Then, the aforementioned plankton net was placed in the flume for ≥10 cycles to collect all surviving prey, which were then counted under a dissecting microscope. The number of prey items captured by the garden eel was estimated by subtracting the number collected from the number released. Control experiments with no garden eels in the flume were replicated 3 times for each combination of flow speed and prey density and showed ≤5% loss of prey. The average loss under each combination of flow speed and prey density in control experiments was subtracted from our estimate of the number of prey items captured by the eel in the corresponding combination. The adjusted number of prey items captured was divided by the time of one water cycle to yield feeding rate (number of nauplii min^−1^).

### 3D reconstruction of eel posture

We recorded the experiments using two iPhone 6s and an iPhone 7 at 60 frames s^−1^ with a resolution of 1920×1080 pixels to reconstruct body postures in 3D using direct linear transformation (DLT). The three cameras were positioned in a triangular arrangement, on one side of the flume, with two side by side and the third above the other two, all focused on the garden eel. Records covered the full duration of each trial. The three videos from each trial were synchronized with a flashing laser pointer.

To reconstruct garden eel posture in 3D from the 2D images of the three cameras, the cameras were first calibrated intrinsically and extrinsically. Intrinsic calibration concerned camera properties that were independent of the camera position, such as the focal length, image size and the location of the principal point of each camera, and was performed using the OpenCV package in Python (https://opencv.org/) with videos of a checkerboard moving toward the camera. Extrinsic properties involve the position and orientation of the cameras and were estimated with the Matlab package easyWand5, which returns DLT coefficients (Theriault et al., 2014). To perform extrinsic calibration, easyWand5 requires videos with a wand swinging over the field of view, a set of pictures of dotted grid paper, a set of pictures of a box with markers specifying the origin, the *x*-axis, the *y*-axis and the *z*-axis in the flume, and the intrinsic properties of each camera. The *x*-axis was defined as the streamwise direction with the upstream direction designated as positive. The *y*-axis was defined as the lateral direction. The *z*-axis was perpendicular to the sandy bed with positive upward. The origin of the coordinate system was placed at the burrow of the garden eel (Fig. 1). Using DLT coefficients obtained from the extrinsic calibration, 2D coordinates from the videos were transformed into 3D coordinates (Hartley and Zisserman, 2004).

The Python package DeepLabCut was used to digitize body parts of garden eels (Mathis et al., 2018; Nath et al., 2019). We manually digitized images extracted from experimental videos to train a deep neural network to automatically digitize two points on the garden eels, the eye and the first large black spot closest to the eye. Experimental videos from the three cameras were digitized separately in DeepLabCut by the Deigo high-performance computing center at the Okinawa Institute of Science and Technology. 2D coordinates of body features were then transformed into 3D coordinates with DLT coefficients (Movie 1; Hedrick, 2008). Digitized positions of the eye were then plotted in polar coordinates to measure eel movements during foraging and strikes under different prey densities and flow speeds.

### Analysis of foraging movements

A strike was defined as an open-mouthed lunge toward a prey. For each strike, the time and location of its initiation were defined as the point at which the eel started to move toward the prey. The point was clearly visible in video records. Foraging parameters were defined as follows. Strike time: the duration (in s) between strike initiation and prey capture. Strike distance: the distance (in cm) between the eel's eye at strike initiation and at prey capture. Strike speed: strike distance divided by strike time (cm s^−1^). Reactive distance: the distance (in cm) between the eel's eye and the prey at the instant of strike initiation. These parameters were computed using the Earth's (flume's) frame of reference based on the duration of strikes and 3D coordinates of the head, which were approximated from the location of the eye. Strike speed, in particular, was also computed using the water frame of reference taking flow speed into consideration. To obtain the reactive distance in each strike, the location of the prey at the initiation of a strike had to be estimated. Given that *Artemia* nauplii are relatively poor swimmers (Trager et al., 1994), we assumed that they would travel straight in the streamwise direction (*x*-axis). Thus, *x*-coordinates of detected prey were estimated by the distance the prey traveled along the *x*-axis during strike time, and *y-* and *z*-coordinates of detected prey were approximated with the coordinates captured. Ten strikes for each trial were used to measure the above parameters, as well as the successful strike rate, defined as the number of prey items captured during a trial divided by the corresponding number of strikes for each of the aforementioned combinations of prey density and flow speed.

### Estimation of vectorial dynamic body acceleration

To estimate trends in energy consumption, we examined the vectorial dynamic body acceleration (VeDBA), which is strongly correlated with oxygen consumption (Ladds et al., 2017; Qasem et al., 2012; Wilson et al., 2006; Wright et al., 2014). VeDBA was calculated as:
(2)

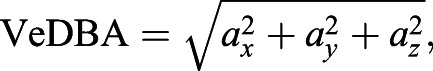
where *a_x_*, *a_y_* and *a_z_* are dynamic body acceleration values measured in three orthogonal axes in the Earth's frame of reference. Because of the slenderness of garden eels, we used 3D reconstructed movements of garden eels instead of attaching accelerometers to them, as in previous studies. The acceleration of the first black spot was used to compute VeDBA, because it was the closest spot to the center of the body that can be seen clearly throughout the trials. With the first black spot position time series, we employed the second-order forward finite difference method to obtain instantaneous acceleration in each axis. The sampling rate was 60 Hz, the same as the frame rate of the videos. We compared the temporal average of raw VeDBA values among three individuals under different flow speeds and prey densities.

### Estimation of drag force and drag coefficient

To compare the drag coefficient and drag force exerted on garden eels at different flow speeds, we used the method of Khrizman et al. (2018). In short, we identified six or seven body points manually and divided the garden eel into seven or eight segments based on the points in 3D. Treating each segment as a cylinder, with a diameter of 0.5 cm, the drag coefficient and drag force were estimated for 10 frames at flow speeds of 0.10 and 0.25 m s^−1^ at a prey density of 1000 m^−3^ for individuals A, B and C. In each frame, the drag force exerted on the whole body outside of the burrow was calculated as the sum of the drag force on each segment. From the total drag force, we estimated the drag coefficient of the whole body.

At different flow speeds, body posture and length are the main differences. More bending and shorter lengths are seen at higher flow speeds. Thus, to understand how much each factor contributes, we also simulated drag coefficients and drag forces in two scenarios: (1) garden eels maintain the posture and length seen at 0.10 m s^−1^, even at 0.25 m s^−1^, and (2) garden eels shorten the length, but keep the straight posture seen at 0.10 m s^−1^, even at 0.25 m s^−1^.

### Statistical analysis

For statistical analysis throughout this study, we used a linear mixed model fitted by REML (restricted maximum likelihood) that can treat the random effects of dependent data. Data were analyzed by specifying prey density or/and flow speed as fixed effects and individual as a random intercept effect using the lme4 package (Bates et al., 2015) in R (http://www.R-project.org/). Significance was computed with the lmerTest package (Kuznetsova et al., 2017), which performs analysis of variance to acquire *P*-values by applying the Kenward–Roger's degrees of freedom method for mixed models. Similarly, the significance for multiple comparisons was computed with the lsmeans package (Lenth, 2016), which obtains least-square means and computes *P*-values adjusted with the Tukey *post hoc* test, using the Kenward–Roger's degrees of freedom method for contrasts.

In detail, the effects of prey density and flow speed on feeding rate were modeled with prey density and flow speed as fixed effects and individual as a random effect. Effects of prey density on feeding rate at each flow speed were modeled with prey density as a fixed effect and individual as a random effect, and linear regression was fitted to the fixed effect. Foraging parameters, VeDBA and successful strike rate at a prey density of 1000 m^−3^ were modeled with flow speed as a fixed effect and individual as a random effect.

## RESULTS

**Fig. 2.**
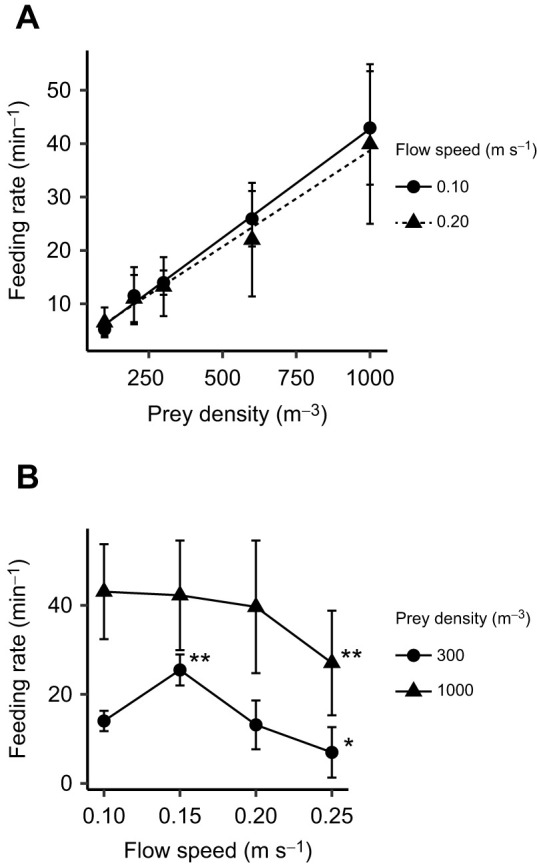
**Feeding rate as a function of prey density and flow speed.** (A) Feeding rate versus prey density at a flow speed of 0.10 or 0.20 m s^−1^ (linear regression by mixed model). (B) Feeding rate versus flow speed at a prey density of 300 or 1000 m^−3^ (mixed model, *F*_3,12_=28.24, *P*<0.01 at 300 m^−3^; *F*_3,12_=8.30, *P*<0.01 at 1000 m^−3^). Data for each individual were averaged from two trials in each condition. Values are means±s.d. among individuals (*n*=5 individuals). Significance, adjusted with the Tukey *post hoc* test, is indicated by asterisks (*P<0.05 and **P<0.01 compared with values at 0.10 m s^−1^).

Under conditions of variable prey density and fixed flow speed (design I), feeding rate increased significantly (mixed model, *F*_4,36_=61.76, *P*<0.01) with increasing prey density (Fig. 2A), with trends exhibiting a nearly linear functional response. The increase in prey flux due to increasing flow speed from 0.10 to 0.20 m s^−1^ had no significant effect on the eel's feeding rate (mixed model, *F*_1,36_=0.75, *P*=0.39). Under conditions of fixed prey density and increasing flow speed (design II), feeding rate did not decrease up to 0.20 m s^−1^, but then dropped at 0.25 m s^−1^ compared with values at 0.10 m s^−1^ (mixed model, *F*_3,12_=28.24, *P*<0.01 at 300 m^−3^; *F*_3,12_=8.30, *P*<0.01 at 1000 m^−3^; *P*=0.02 and *P*<0.01 at 300 and 1000 m^−3^, respectively, adjusted with the Tukey *post hoc* test to compare values between 0.10 and 0.20 m s^−1^). Eels retreated into their burrows at flow speeds exceeding 0.30–0.35 m s^−1^. Occasionally, eels protruded only their snouts under strong flow speeds, but did not attempt to feed. The absence of a positive effect of increasing flow speed was accentuated as a result of its linear contribution to the flux of prey, suggesting the occurrence of other flow-related effects.

**Fig. 3.**
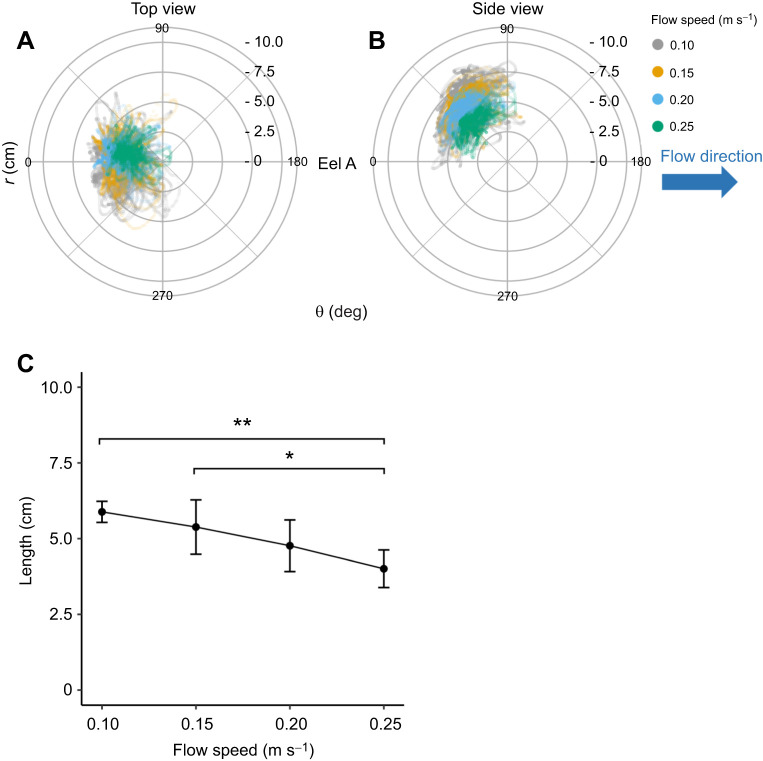
**Reduction of movement flexibility and body length outside the burrow with increased flow speed.** (A,B) Position of an eel's eye during experiments. Each point indicates the position of the eye of a representative eel (eel A) in polar coordinates with the burrow as the origin, as seen from (A) above and (B) the side, under four flow speeds (0.10, 0.15, 0.20 and 0.25 m s^−1^) and a fixed density of prey (1000 m^−3^). Positions are plotted for the entire experimental duration for two trials at each flow speed for the individual (6720, 4480, 3360 and 2688 frames for 0.10, 0.15, 0.20 and 0.25 m s^−1^, respectively). (C) Body length outside the burrow at different flow speeds at a prey density of 1000 m^−3^. Data for each individual were averaged from the entire experimental duration for two trials at each flow speed. Values are means±s.d. among individuals (*n*=3 individuals; *F*_3,6_=9.73, *P*=0.01). Significance, adjusted with the Tukey *post hoc* test, is indicated by asterisks (*P<0.05 and **P<0.01).

3D reconstruction of eel movements indicated that the portion of an eel's body out of the burrow and the extent of its movement decreased with increasing flow speed (Fig. 3; mixed model, *F*_3,6_=9.73, *P*=0.01).

**Fig. 4.**
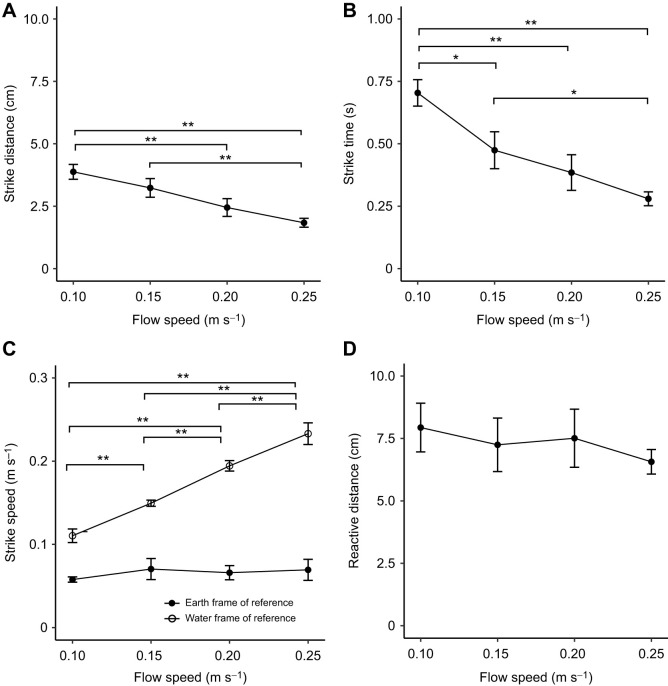
**Foraging parameters at different flow speeds.** (A) Strike distance, (B) strike time, (C) strike speed and (D) reactive distance (see Materials and Methods) of garden eels at different flow speeds at a prey density of 1000 m^−3^. Data for each individual were averaged from 20 strikes at each flow speed. Values are means±s.d. among individuals (*n*=3 individuals; mixed model, A: *F*_3,6_=24.87, *P*<0.001; B: *F*_3,6_=30.13, *P*<0.001; C: *F*_3,6_=1.05, *P*=0.44 for earth frame of reference, *F*_3,6_=222.9, *P*<0.001 for water frame of reference; D: *F*_3,6_=1.07, *P*=0.43). Significance, adjusted with the Tukey *post hoc* test, is indicated by asterisks (*P<0.05 and **P<0.01).

**Fig. 5.**
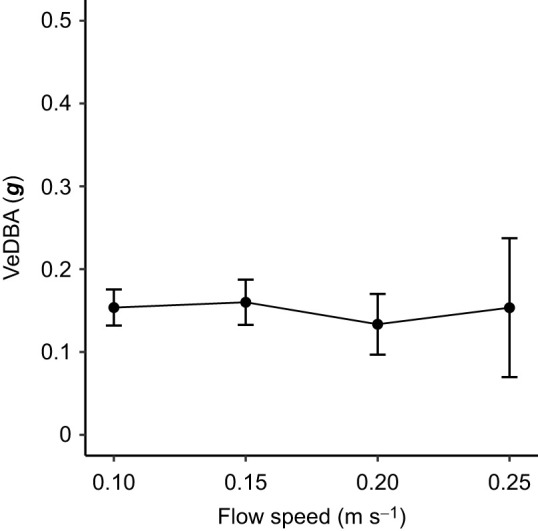
**Vectorial dynamic body acceleration (VeDBA) at different flow speeds.** Data for each individual were averaged over all frames during trials for each flow speed. Values are means±s.d. among individuals (*n*=3 individuals; mixed model, *F*_3,6_=0.45, *P*=0.72).

Strike distance and time significantly decreased as flow speed increased (Fig. 4A,B; mixed model, *F*_3,6_=24.87, *P*<0.001; *F*_3,6_=30.13, *P*<0.001), while the corresponding strike speed and reactive distance showed no significant change (Fig. 4C,D; mixed model, *F*_3,6_=1.05, *P*=0.44; *F*_3,6_=1.07, *P*=0.43). The estimated energy expenditure on behavior at different flow speeds (VeDBA) showed no significant effect of flow speed (Fig. 5; mixed model, *F*_3,6_=0.45, *P*=0.72).

The drag coefficient of the actual posture dropped 23.8% at 0.25 m s^−1^ compared with that at 0.10 m s^−1^. Neither simulated drag coefficient differed significantly from the value at 0.10 m s^−1^ (Fig. S1A). Although the higher flow speed increased the drag force 2.6 times in actual experiments, it would have increased 6.0 times if the eel did not change its posture and length (scenario 1; Fig. S1B). From the simulated posture and length, changes in length and posture contribute 28.4% and 40.0% decreases in the drag force, respectively. Together, the bending posture and shortened length at high flow reduced the drag force by 56.8%, compared with the simulated drag force at the same flow assuming a straight posture and the same length observed at low flow.

**Fig. 6.**
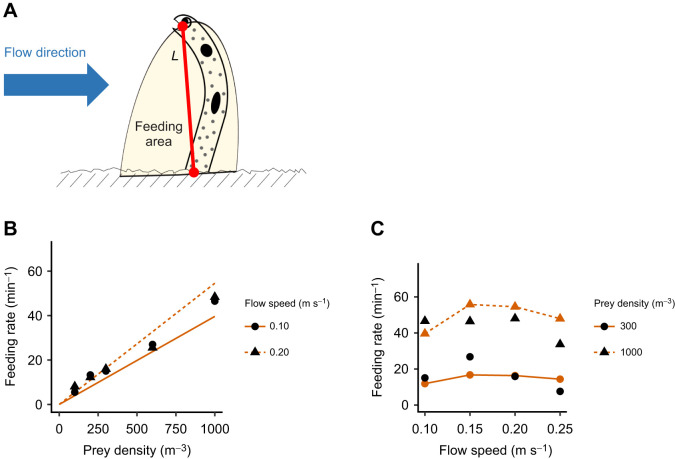
**Foraging model.** (A) Schematic drawing that describes the foraging model. *L* is the third quartile of length out of the burrow at 1000 m^−3^. (B,C) Feeding rate as a function of (B) prey density (at a flow speed of 0.10 or 0.20 m s^−1^) and (C) flow speed (at a prey density of 300 or 1000 m^−3^). Solid black circles and triangles indicate the empirical mean feeding rate from the experiment. Red lines, circles and triangles indicate the feeding rate estimated by the model.

Feeding rate and the length of the body out of the burrow decreased as flow speed increased (Figs 2B and 3C). Thus, we modeled garden eel foraging assuming that the length out of the burrow is a key factor governing feeding behavior. The successful strike rate was unaffected by flow speed (mixed model, *F*_3,6_=1.68, *P*=0.47) and ranged from 0.95 to 1.30 at a prey density of 1000 m^−3^ for all flow speeds tested. Considering the high successful strike rate and short handling time, we assumed that garden eels can capture all prey that pass through a semi-circular feeding area, the radius of which is approximated as the third quartile of the eel's length out of the burrow at a specific flow speed (Fig. 6A). As a radius, the third quartile was used as a value between the maximum and the average, because the maximum length circumscribes an area larger than that used by the eels while the average does not encompass the entire area covered by the eels. In the proposed model, the feeding rate *F* was estimated as:
(3)

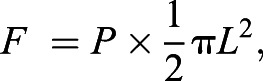
where *P* is prey flux and *L* is the third quartile of the length out of the burrow. The proposed model agreed well with experimental results (Fig. 6B,C).

## DISCUSSION

### Prey density

Garden eels are unique fish that feed while anchored in a semi-permanent burrow. This restricted lifestyles make garden eels adapt to particular environmental regimes and therefore studying their interactions with the environment is valuable. The positive correlation between feeding rate and prey density was nearly linear, and the observed minimum interval between strikes was around 0.3 s, suggesting that handling time is negligible in the prey density range tested. Our experiments showed a fit to the linear part of a type II functional response (Holling, 1959).

### Currents and energetic cost–benefit

Flow speed is expected to modulate foraging because at increased flow speed fish movement is restricted and more energy is required for locomotion, while there is a simultaneous increase in prey flux (Fausch, 1984; Kiflawi and Genin, 1997). Garden eels did not show a monotonic increase in feeding rate as flow speed increased. At a specific prey density, garden eels protruded less and exhibited more restricted motion as flow speed increased. At flow speeds ≤0.20 m s^−1^, feeding rate showed no significant change at a prey density of 1000 m^−3^, suggesting that suppression of movement offset the effect of increased prey flux. From the 3D analysis, at higher flow speed, garden eels modified their strikes to focus on closer prey. Although their body posture is not exactly the same as that of garden eels in the Red Sea (Khrizman et al., 2018), the bending posture and shorter length at higher flows reduced the drag force by 56.8% compared to the simulated drag force at the same flow assuming a straight posture and the length observed at low flow (Fig. S1). Modification of behavior and posture allows garden eels to maintain energy expenditure at a similar level to that at flow speeds ≤0.20 m s^−1^. At 0.25 m s^−1^, the feeding rate dropped by more than 30% compared with that at lower flow speeds, because the limitation of restricted movement became more significant than the benefit of increased prey delivery. At all flow speeds, garden eels continuously showed active strikes rather than assuming a static posture dominated by isometric contraction, making the VeDBA valuable as a proxy for relative energy expenditure (Movie 1). Stable VeDBA values at all flow speeds suggest that garden eels can maintain a level of energy expenditure by adjusting the length out of the burrow and their posture. For all flow speeds examined, garden eels showed indistinguishable changes in strike speed. In drift-feeding river fish that also show a constant strike speed, strike speed was close to the predicted maximum sustainable swimming speed, helping those fish to minimize handling time and avoid burst swimming, which generates oxygen debt (Piccolo et al., 2008; Puckett and Dill, 1984). Similarly, the constant strike speed of garden eels under all tested flow speeds can be interpreted in terms of maintaining energy expenditure. When the flow speed exceeded 0.30–0.35 m s^−1^, garden eels retracted into the burrow. Because the feeding rate may even decrease at these fast flows, based on our results, eels may have retracted because the energy expenditure during feeding exceeds the benefit of feeding.

The trend in feeding rate as a function of flow speed is similar to that in planktivorous reef fish. However, garden eel feeding is adapted to higher flows because the peak occurred just below 0.20 m s^−1^ in our experiments, whereas for most reef fish it occurs below 0.15 m s^−1^ (Clarke et al., 2009; Kiflawi and Genin, 1997), allowing garden eels to live in relatively exposed sandy areas. Free-swimming fish in coral reefs or rivers have access to shelters that can reduce currents by more than 60%, conserving energy for locomotion and foraging (Johansen et al., 2007, 2008; Taguchi and Liao, 2011). As garden eels live without shelters other than their burrows, to acquire food, they need to adapt to higher flows compared with free-swimming fish in coral reefs. River fish either seek refuge without foraging or forage in the stream at high flow, because fish consume the most energy when moving across a strong velocity gradient (Johansen et al., 2020). By adapting to higher flows, garden eels may reduce the necessity to traverse strong velocity gradients. Using the trend of feeding rates and VeDBA, we can estimate the energetic cost and benefit curve for garden eels. According to a study on teleosts, VeDBA correlated with oxygen consumption and increased exponentially with flow speed (Wright et al., 2014). Although VeDBA in anchored fish has not been shown to correlate with oxygen consumption, use of VeDBA is so far the best available way to estimate relative energy expenditure at different flow speeds using precisely measured 3D movement data. Cost–benefit curves of fish have an optimum range in which fish maximize net energy gain (Grossman, 2014; Hill and Grossman, 1993). For garden eels, the optimal flow speed approximated by feeding rate and VeDBA was 0.10–0.20 m s^−1^ (Figs 2B and 5). In summary, garden eels benefit most from feeding in currents at 0.10–0.20 m s^−1^. They continue to accrue benefits from feeding up to a flow speed of 0.30–0.35 m s^−1^, but at declining levels, and all benefits cease in stronger flows. Eels occasionally extend the tips of their snouts slightly to sense the flow at these strong flow speeds. Because flow is one of the most important requirements for aquatic animals, our findings help identify critical habitats for garden eels. To precisely assess energy expenditure, further follow-up studies employing more direct measurement of energy costs, such as oxygen consumption, are needed for anchored fish.

### Foraging model

The disk equation is a classic way to predict foraging. Recently, foraging models have considered prey flux, rather than just prey density. Especially for aquatic animals, flow speed is an important factor with regard to the prey encounter rate and the energetic cost of locomotion (Fausch, 1984; Kiflawi and Genin, 1997). Usually, foraging models of fish are based on the reactive volume, a wedge calculated from empirical reactive distance and angle. Although the range of flow speed differs, both drift-feeding river fish and coral reef fish narrow the feeding area mainly in lateral or streamwise directions as flow speed increases (Kiflawi and Genin, 1997; O'Brien and Showalter, 1993; Piccolo et al., 2008). Fish narrow the angle to avoid being oriented lateral to the flow with the risk being swept down-current. However, garden eels did not change the reactive distance as flow speed increased, suggesting their ability to detect prey was not affected by flow speed. In contrast, movements were affected by stronger flow to focus on a smaller search area or volume, as their strike distance and length out of the burrow decreased with increasing flow speed. Thus, we assumed that their foraging area can be approximated as the area of the semicircle defined by the length out of the burrow. Feeding rates from the proposed foraging model closely match experimental data. Note that the model showed a higher feeding rate under high flow speeds than our observed values. Several foraging models attributed overestimated feeding rates to decreased prey detection probability, which is challenging to integrate into models (Hughes et al., 2003; Kiflawi and Genin, 1997). If search volume is simply assumed to be a cylinder or wedge dependent on detection area and reactive distance, prey detection probability decreases for fixed detection area and reactive distance where a velocity-dependent increase in the search volume occurs (Piccolo et al., 2008). However, in our experiments, although garden eels did not change their reactive distance as flow speed changed, they may have overcome the prey detection limitation by protruding less and focusing on a smaller feeding area. Overestimation may have resulted instead from garden eels being unable to reach some parts of the semicircle if the extended body length exceeds a certain threshold at high flow speed. Thus, the model could be improved by factoring in specific angles or regions that garden eels cannot reach, although this is difficult to incorporate and complicates the model.

### Comparison between garden eels

The feeding behavior of the spotted garden eel, *Heteroconger hassi*, differs from that of the Red Sea species *Gorgasia sillneri* in several respects. While the Red Sea species showed a monotonic increase in feeding rate with increasing flow speed (Khrizman et al., 2018), the spotted garden eel did not. The difference may be due to experimental conditions or interspecific differences. Experiments in the field have the advantage of realistic conditions, while laboratory experiments allow more control over factors that can influence results. *In situ* feeding is affected by environmental factors such as illumination, turbidity, temperature, debris and prey-specific escape behavior (Hill and Grossman, 1993; Howard and Bori, 1972; Manatunge and Asaeda, 1998; O'Brien and Showalter, 1993; Rickel and Genin, 2005), all of which were fixed in our flume trials. Khrizman et al. (2018) estimated feeding rates by counting bites seen in video records, rather than by directly measuring feeding rate, as in our experiment. As resuspension of debris over shallow bottoms is expected to increase with increasing flow speed (McCave et al., 1995), more abundant debris *in situ* at higher flows could bias estimates of feeding rate. Note that in the flume, we observed several cases in which the garden eels captured non-edible particles larger than *Artemia* nauplii that were immediately expelled.

These two species belong to different genera that differ morphologically. *Gorgasia* has a longer gape, a finless tail and fewer rows of teeth, suggesting a different feeding behavior from that of *Heteroconger* (Böhlke, 1957). Recent studies also mentioned differences in the composition of skin cells or reproductive behavior between *Heteroconger* and *Gorgasia* (Canei et al., 2020; Kakizaki et al., 2015). The difference in body posture under high flow speeds may result from phylogenetic differences, possibly affecting the degree of decrease in drag force, which is greater for *Gorgasia* and less for *Heteroconger*. One explanation for the different responses in feeding behavior is the length of garden eels (18–30 cm for *H. hassi* in our experiments, versus 55–95 cm for *G. sillneri*). Because of their shorter and more slender bodies, and thus the smaller amount of muscle, spotted garden eels may be unable to maintain feeding under strong flows, as *G. sillneri* does. Interestingly, we observed that spotted garden eels exhibit similar body posture to that of *G. sillneri* only when they defecated at flow speeds of 0.20 m s^−1^. Further research is required to ascertain the reason for differences in feeding behavior in these two garden eel species.

Garden eels cannot adopt the strategies for strong flows that free-swimming fish employ, such as changing proportions of fin type usage or adopting sheltering behavior, suggesting that garden eels have different foraging strategies for flow. By isolating and evaluating the effects of prey density and flow speed on feeding behavior of garden eels, our study revealed their unique strategy to adapt to live in relatively exposed sandy areas with reduced competition from other planktivorous fish. Modulated movements to counter changes in flow speed enable the eels to sustain feeding under a wide range of flow conditions. To our knowledge, only the study on *G. sillneri* in the Red Sea (Khrizman et al., 2018) and this study discuss feeding behavior of garden eels and its modification in relation to environmental factors. To discuss their responses in more realistic environments, further studies must evaluate the effects of other environmental factors such as turbidity, prey size and composition, and more complex flow conditions.

## Supplementary Material

Supplementary information
