# Supplementary material for: Effects of prey density and flow speed on plankton feeding by garden eels: a flume study
Source: J Exp Biol. 2022 Apr 22;225(8):jeb243655. doi: 10.1242/jeb.243655 (PMC9124482; doi:10.1242/jeb.243655)
Supplement: Supplementary information [file jexbio-225-243655-s1.pdf]

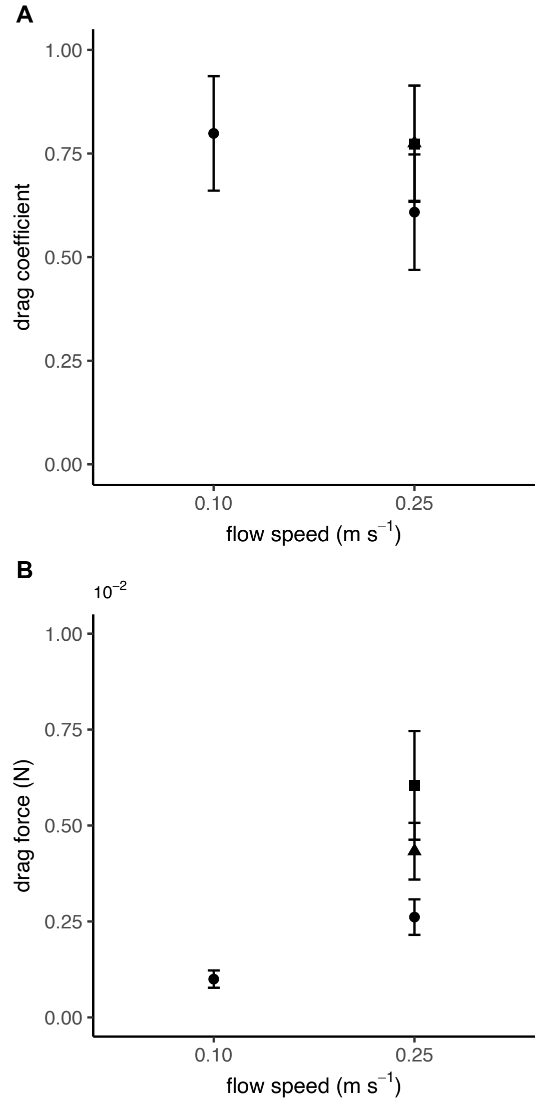

Fig. S1. Drag coefficient and drag force. Data for each individual were averaged from ten frames at each flow speed. Values are means ± s.d. among individuals (*n*=3 individuals). Circles are from experimental measurements. Squares simulate values at 0.25 m s^{-1} assuming the length and the posture are the same as those at 0.10 m s^{-1}. Triangles simulate values at 0.25 m s^{-1} assuming the posture remains the same as that at 0.10 m s^{-1} while maintaining the same exposed length at 0.25 m s^{-1}.

Journal of Experimental Biology • Supplementary information

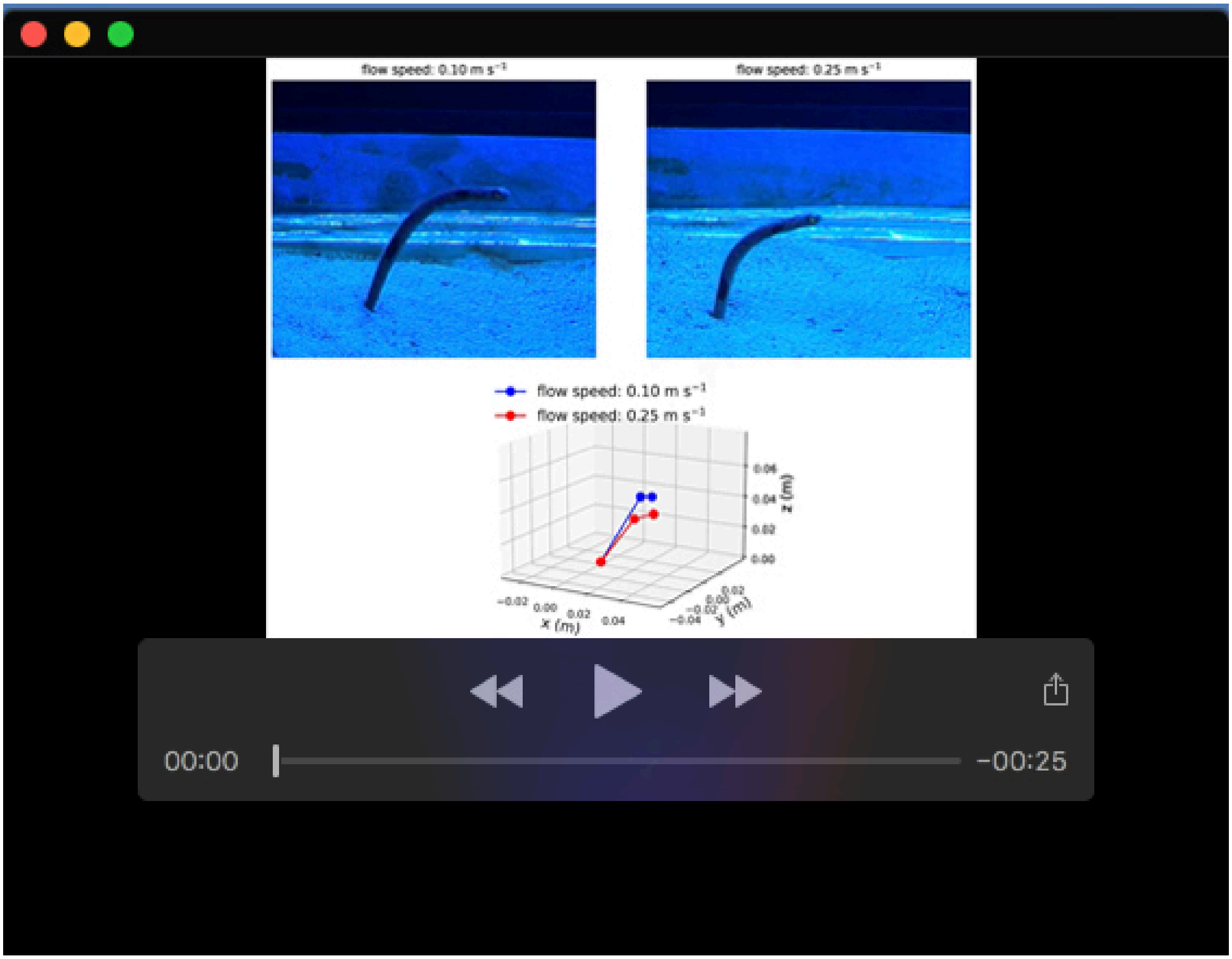

Movie 1. 3D reconstructed garden eel movement. Upper left and right panels show the videos taken during experiments at flow speeds of 0.10 and 0.25 m s^{-1}, respectively. Blue and red lines in bottom panel show the corresponding 3D reconstructed eel movements at flow speeds of 0.10 and 0.25 m s^{-1}, respectively.
